# An open-source digital contact tracing system tailored to haulage

**DOI:** 10.3389/fdgth.2023.1199635

**Published:** 2023-07-19

**Authors:** Adrian Muwonge, Bryan A. Wee, Ibrahimm Mugerwa, Emma Nabunya, Christine M. Mpyangu, Barend M. de C. Bronsvoort, Emmanuel Robert Ssebaggala, Aggelos Kiayias, Erisa Mwaka, Moses Joloba

**Affiliations:** ^1^ Digital One Health Laboratory, The Roslin Institute, College of Medicine and Veterinary Medicine, University of Edinburgh, Edinburgh, United Kingdom; ^2^ Blockchain Technology Laboratory, School of Informatics, University of Edinburgh, Edinburgh, United Kingdom; ^3^ Ministry of Health, Kampala, Uganda; ^4^ School of Biomedical Sciences, College of Health Sciences, Makerere University, Kampala, Uganda; ^5^ College of Humanities and Social Sciences, Makerere University, Kampala, Uganda; ^6^ Bodastage Solutions, Kampala, Uganda; ^7^ Department of Anatomy, College of Health Sciences, Makerere University, Kampala, Uganda

**Keywords:** targeted digital, contact tracing, COVID-19, global south, LMIC, open-source

## Abstract

Digital contact tracing presents numerous advantages compared to manual contact tracing methods, especially in terms of enhanced speed and automation. Nevertheless, a lack of comprehensive evaluation regarding functionality, efficiency, benefits, and acceptance within communities remains. Here we primarily focus on the functionality of THEA-GS, an open-source digital contact tracing tool developed through consultation with stakeholders. Additionally, we provide insights from its implementation on a limited sample of haulage drivers in Uganda, serving as a representative case for a low- and middle-income country. THEA-GS comprises two primary components: (a) a smartphone application, and (b) a suite of server-programs responsible for data processing and analysis, including databases and a web-based interface featuring dashboards. In essence, the mobile application records the timestamped location of haulage drivers within the road network and identifies possible transmission hotspots by analyzing factors such as the duration of stops and the communities associated with them. The tool can be integrated with national infrastructure to compare drivers’ diagnostic results and contact structure, thereby generating individual and community risk assessments relative to the road network. During the Omicron-variant wave of the COVID-19 pandemic, a total of 3,270 haulage drivers were enrolled between October 2021 and October 2022. Around 75% of these drivers utilized THEA-GS for approximately two months. Based on an analysis of 3,800 test results, which included 48 positive cases, 125 contacts, and 40 million time-stamped GPS points, THEA-GS shows a significant speed improvement, being approximately 90 times faster than MCT. For instance, the average time from sample collection to notifying a case and their contacts was approximately 70 and 80 min, respectively. The adoption of this tool encountered challenges, mainly due to drivers’ awareness of its purpose and benefits for public health. THEA-GS is a place-based digital contact tracing tool specifically designed to assist National Public Health Institutions in managing infectious disease outbreaks involving the haulage industry as a high-risk group. While its utility, acceptance, and accuracy have not been fully evaluated, our preliminary tests conducted in Uganda indicate the tool’s functionality is robust, but social acceptance and adoption are heavily reliant on establishing trust among users.

## 1. Introduction

The World Health Organization (WHO) has outlined a digital health strategy that emphasizes the need for sustained national infrastructure investment to ensure equitable, affordable, and universal access to eHealth (1). While this strategy primarily focuses on primary healthcare, the same principle should apply to any future digital public health strategy. The response to the COVID-19 pandemic has attracted an unprecedented influx of investment in digital health innovations, such as digital contact tracing (DCT), which has been employed alongside other non-pharmaceutical interventions to mitigate the transmission of the virus (1–3).

DCT aims to automate the processes involved in manual contact tracing (MCT), a traditional public health tool used for controlling the spread of infections between individuals (4). MCT typically involves several key steps: (a) identification and notification of cases, (b) identification of contacts by compiling a list of individuals who have had close interactions with the identified cases, (c) notifying the identified contacts by providing them with official public health guidance via phone calls, and (d) conducting follow-ups with the contacts through visits or phone calls to monitor their health status during a specified isolation period (4). These steps make MCT a labour-intensive and costly approach. For instance, during the COVID-19 pandemic, it was estimated that the United States and the Philippines needed approximately 100,000 and 250,000 contact tracers respectively, which would have cost $350 million and $90 million per month (5).

The effectiveness of manual contact tracing (MCT) in public health is contingent upon its ability to outpace disease outbreaks (4, 5). This is why its utility was significantly diminished by a fast-paced outbreak like COVID-19. To address this, the widespread use of DCT was used in many countries to expedite contact identification and notification (4, 5). The utility of DCT can improve efficiency particularly when directed towards a specific group (6). This is why its use to target long-distance cross-border haulage truck drivers and their assistants was viewed as a potential solution to enhance contact tracing for infectious diseases (7, 8).

During the COVID-19 pandemic, haulage drivers continued to travel long distances to maintain supply chains while the general population was under lockdown (7), creating a potential risk for disease transmission across vast geographical areas. In response, public health systems increased testing frequency and implemented targeted contact tracing measures. However, these interventions led to long queues at border checkpoints, placing strain on the supply chains of essential goods such as food, medicine, fuel, and agricultural supplies, as well as adversely affecting the welfare and health of the drivers (9). By harnessing targeted DCT, the impact on supply chains during infectious outbreaks could be minimized. However, countries must develop, adopt, adapt, and test such digital tools. Here, we primarily report on the functionality of THEA-GS. We have also tested its use on a limited population of haulage truck drivers to examine the challenges of uptake. Our motivation is to facilitate universal access to DCT tools, promote transparent implementation, and ensure ethical use and oversight. Therefore in this study, our primary focus is on the functionality of THEA-GS, an open-source DCT tool developed through consultation with stakeholders. Additionally, we test it on a limited sample of haulage drivers in Uganda, serving as a representative case for a low- and middle-income country (LMIC).

## 2. Methods

### 2.1. THEA-GS architecture

**Figure 1:**
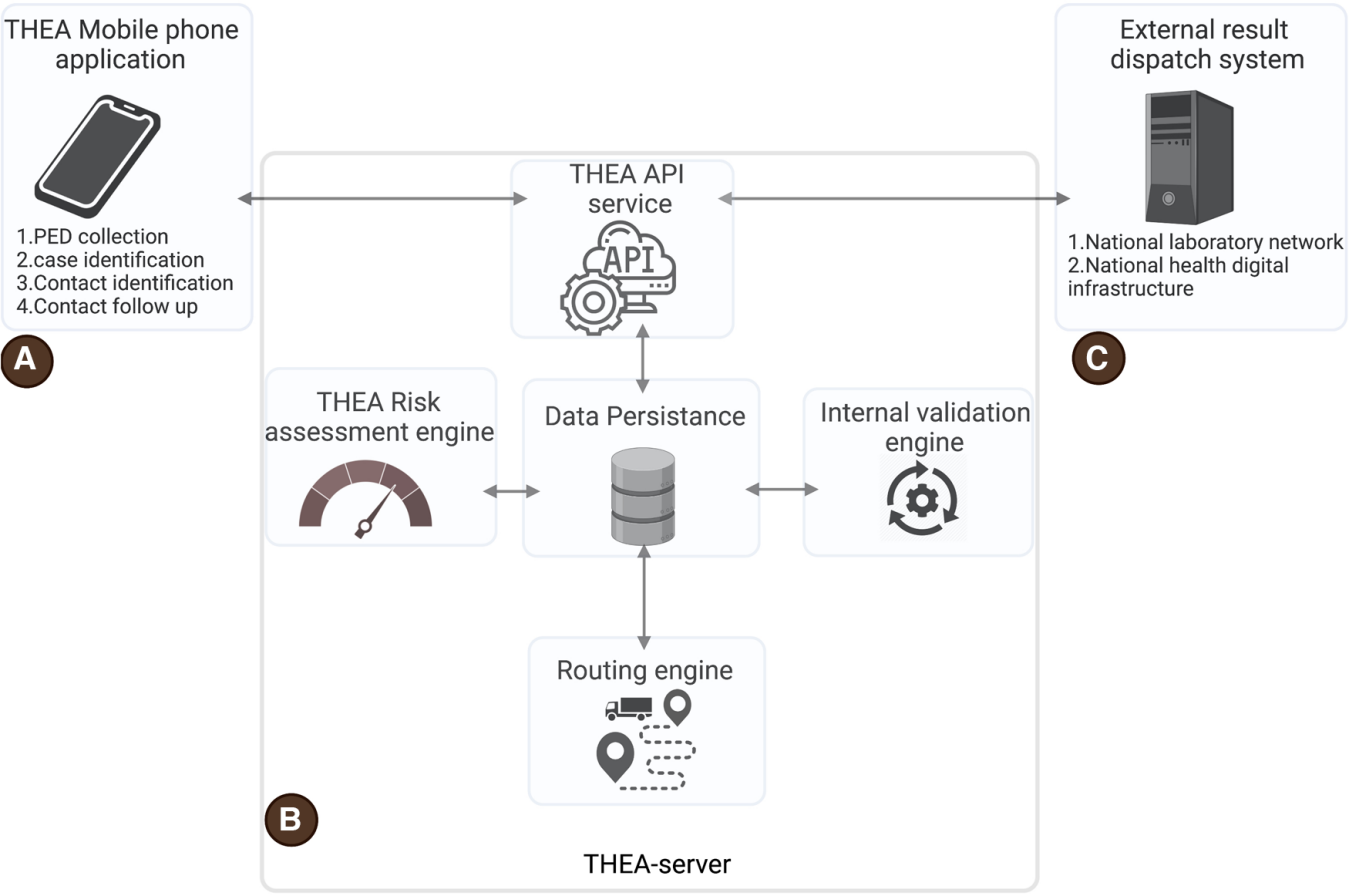
Shows the architecture of the THEA-GS contact tracing system. (**A**) is the THEA-GS mobile application, (**B**) is the THEA-GS server, and (**C**) is the external data feed from the result dispatch infrastructure of a given country. PED is the proximity estimation data which includes timestamped GPS data.

THEA-GS consists of two primary components: (a) a mobile phone application initially available for Android smartphones, and (b) a backend server that receives tracking data and diagnostic results from a national result dispatch system (RDS). The server and a user-friendly web portal can be configured and customized to suit the requirements of a specific country (Figure 1 and Table 1). Both components of THEA-GS are freely downloadable for public health institutions seeking to implement haulage tracking. In addition, detailed documentation can be accessed at the project website: https://project-thea.github.io/thea-gs/. The source code is openly provided under the Apache License, Version 2.0.

**Table 1:** Institutional and individual system requirements for the THEA-GS server and mobile application.

Systems	Components	System requirements	Infrastructure requirements
Mobile application^a^	Mobile app (Android)	Android v5 or higher	7–47 MB
THEA-Server^a^	API service	Dependencies can be installed with Docker. PHP 8+, Vue3, Laravel Web framework 8+, MySQL 8, Jetstream with Inertial, Redis, leaflet, Valhalla. Recommended OS: Linux (but also available for Windows and MacOS)	A central processing unit (CPU), at least 16 GB of random access memory (RAM) & storage of 250GB
	Risk Assessment engine		
	Internal Validation Engine		
	Routing engine		

^a^ Mobile application & THEA-Server.

### 2.2. THEA-GS mobile phone application

The application primarily records timestamped Global Positioning System (GPS) data, which is utilized in the proximity estimation process outlined in the functionality section below. It also stores notifications of the most recent test results and collates users’ testing history by communicating with testing laboratory servers via API as described below. The application offers a personalized exposure risk assessment, along with risk evaluations for popular locations along the road network. Upon downloading the application, drivers have the option to configure it to tailor contact tracing to a specific disease. This selection determines the corresponding API service with which the application interacts on the THEA-GS server.

Upon successful installation of the mobile application, it assigns a unique identifier to the driver and generates a unique universal ID (UUID) to ensure the user’s anonymity. The application operates in the background and logs timestamped GPS coordinates exclusively when the driver is on the road, as the tool is geo-fenced to the road network within a range of approximately ±100 meters.

Our unit data log consists of a compressed file transmitted over an encrypted internet connection. The file contains the GPS coordinates (longitude and latitude), date and time, all linked to a UUID4 unique random string. For example: 12.098765, 12.098765, 2021-07-13 23:00, ad61d41f-8860-41b9-ab2c-29f0eb0ffa76. This log file is a UTF-8 encoded string with 75 characters, which amounts to 600 bits or 75 bytes in size. To optimize storage space usage, the tool is designed to synchronize data with the server as frequently as possible. If the mobile app lacks internet connectivity, data synchronization is paused and the user is notified once connectivity is restored. The default frequency of data synchronization is set at 30 min, although it can be adjusted to suit the local data capacity. Similarly, the frequency of GPS sampling is adjustable to preserve battery life. To address gaps in GPS sampling, a routing algorithm called Valhalla is utilized to reconstruct the driver’s path and travel direction. This information can be viewed on the web portal associated with the tool (10). As part of adapting this tool to local context coordinates and shapefiles for the road network must be included.

#### 2.2.1. THEA-GS server

The server consists of four main subcomponents: (a) THEA-GS API Service, (b) data persistence service, (c) Risk assessment engine, (d) internal validation engine, and (e) a routing engine (Figure 1). The API service facilitates communication between the THEA-GS Server and the mobile application. It also allows for integration with external systems such as laboratory management systems and National result dispatch systems. This integration enables the collection and transmission of test results notifications to the THEA-GS mobile application. All communication with the THEA-GS Server and API Service occurs over encrypted connections to ensure data security. The data persistence service is responsible for storing all the platform’s data.

The THEA-GS Server receives proximity estimation data (PED), which consists of logged timestamped GPS data, from the THEA-GS mobile application. Additionally, it receives diagnostic test information (DTI) from a network of government and private public health laboratories via the API service. The frequency at which this data is received can be adjusted by the user to optimize the infrastructure’s performance.

The PED obtained from the mobile application is utilized by the routing engine to enhance the accuracy of routes and movement directions. The routing engine analyzes the PED and determines the closest road segment (11), effectively mapping the road network as the primary layer. This mapped road network is then overlaid with the output from the risk assessment engine. The internal validation engine plays a crucial role in processing the data inputs and generating key performance indicators (KPIs) to evaluate the tool’s performance in contact tracing. These KPIs include (a) Positive prediction value: This indicator calculates the proportion of notified contacts who test positive within 14 days and, (b) Specificity: This indicator measures the proportion of non-notified drivers who remain negative. These KPIs serve as internal validation metrics to assess how effectively the tool implements contact tracing and its overall performance.

#### 2.2.2. Web interface

The web interface serves as a management tool for the THEA-GS Server, providing various functionalities. It presents an anonymized list of drivers who are using the mobile application, along with their associated test history and computed risk assessments. These details are visualized on a map for easy understanding and analysis. The web interface also includes a dedicated tab for internal validation, where the system’s key performance indicators, as described earlier, are displayed. This metric provides an overview of the system’s performance in terms of contact tracing effectiveness. Additionally, the THEA-GS Web portal features a dashboard that enables monitoring of the platform’s performance. The dashboard presents daily statistics such as the number of drivers active on the system and the corresponding number of test results. Moreover, the system allows for the inclusion of custom-built dashboards tailored to specific requirements and needs of different settings (https://demo.project-thea.org/dashboard).

### 2.3. Setting up THEA-Gs

#### 2.3.1. Integration with national digital infrastructure

The THEA-GS server integrates with national digital health infrastructure to support and complement existing disease surveillance tools. It can also integrate with other tools such as WHO’s data collector services https://www.who.int/tools/godata, to enhance outbreak investigations. Through the API service and webhooks, the server can communicate with laboratory networks, enabling the secure collection and relay of test results to the mobile application used by drivers. To ensure data governance and transparency, access to the backend server is limited to authorized individuals within public health institutions. An access log is maintained, visible to users, to ensure accountability and transparency. Our data governance strategies prioritize privacy and compliance with ethical guidelines, in this regard, once downloaded, users are prompted to read and consent to the terms and conditions. Once the consent process is complete, a digital consent form is generated and stored on the backend server. Users have the option to opt out of this service, which will cease the tracking of their movement and collection of their test result information. However, the collected data will be retained until a time point stipulated at the point of setting up the system.

#### 2.3.2. Setting up THEA-Gs and onboarding haulage drivers

THEA-GS is specifically designed for efficient public health use with minimal overheads. The initial step involves installing and configuring a backend server on a computer with internet connectivity to host the necessary databases. Users can then download the smartphone application and set it up to establish communication with the backend server. The development and testing of THEA-GS in Uganda was done in collaboration with academic institutions to ensure a consultative design approach. Additionally, the private sector provided access to diagnostics, with the Ministry of Health’s oversight. Given the potential sensitivity of the collected data, the tool incorporates pseudo-anonymization strategies to ensure the encryption of sensitive information. The data collection process strictly adhered to individual consent as stipulated in the ethical and government approvals.

The deployment of the THEA-GS Server is simplified by running it in a containerized environment. This allows for fast deployments and developer onboarding. Deploying THEA-GS Server requires the steps below:

(1) Download the most recent release of the THEA-GS Server from GitHub (https://githu.com/project-thea/project-thea-server/releases)
(2) Download and install docker from (https://www.docker.com/)
(3) From the command terminal, go to the THEA-GS source folder you downloaded in Step 1.
(4) Use docker-composer to launch THEA-GS Server. docker-composer up –d
(5) Access THEA-GS Server at https://localhost:8000 from your browser

Detailed installation and setup instructions can also be found here.

#### 2.3.3. Technical specifications

To set up THEA-GS on national public health infrastructure, institutions should have a central processing unit (CPU) with a minimum of eight dedicated cores, 16 gigabytes (GB) of random access memory (RAM), and a storage capacity of 250 gigabytes (GB). For the smartphone component, the Android operating system requires approximately 7–47 megabytes (MB) for the installation of the mobile application (Table 1). The architecture of THEA-GS is designed to scale horizontally as the traffic and demand increase. The application programming interface (API) server is configured to handle multiple concurrent requests efficiently. The database server is optimized to prioritize write operations over read operations, ensuring smooth data management. The application server is responsible for hotspot generation and infection prediction, as outlined in subsequent sections.

#### 2.3.4. Security features

The THEA-GS system employs standard security mechanisms to ensure data protection to complement user anonymity. User authentication is implemented using the Laravel Jetstream framework (12). This allows a mix of role-based access control functions and encryption of all communications with the THEA-GS server using transport layer security (TLS) that prevents man-in-the-middle attacks and eavesdropping. All this is done in addition to maintaining access logs and all server access data is for authorized personnel only.

### 2.4. THEA-GS functionality

#### 2.4.1. Proximity estimation and individual risk

Unlike peer-to-peer contact tracing, THEA-GS implements a place-based contact tracing that utilizes time-stamped GPS data for the proximity estimation process, which is further refined by incorporating “ground truth information” (GTI). The GTIs consist of various locations such as fuel stations, designated stops, hospitals, weighing bridges, trailer parks, ports of entry and exit, and detected hotspots. These GTI coordinates are also used as parameters for the risk assessment process. To estimate the proximity between truck drivers, a clustering algorithm is employed, considering factors such as maximum proximity duration (T), minimum occupancy (W), and distance (D) (13, 14). These parameters define the maximum dimensions of a cluster in terms of time, the number of drivers, and physical space, all of which depend on the speed of movement (i.e., velocity) (14, 15). The algorithm initiates by randomly selecting a starting point as the centroid (C) of the first cluster. For each subsequent point within the specified time (T) and distance (D) thresholds, a new cluster centroid is calculated based on the average distance between the previous point and the current data point. This process continues until all points converge, and the cluster centroid stops moving. Any points that fall outside the defined (T) and (D) ranges are not included in the cluster (C) but may fall under an adjacent cluster. The algorithm then identifies clusters with an occupancy greater than (W) as potential hotspots, indicating locations where drivers have a higher likelihood of proximity and potential transmission risks. In Uganda, 20 min was used as T, with the expectation that this parameter would be adjusted using empirical data.

After generating a cluster, the centroid associated with the cluster is cross-referenced with the GTI coordinates. This step helps identify locations that may be linked to potential transmission between drivers and pose a risk to nearby communities. Clusters that are not directly associated with GTIs are then ranked based on their frequency of occurrence. The top-ranking clusters are added to the GTI list as hotspots. When a driver tests positive, their membership in the cluster with the longest duration indicates a higher probability of transmission. This forms the basis of the risk assessment process used to notify drivers who may have been exposed to an infected individual. By evaluating how many of the notified drivers later test positive themselves, the system can iteratively optimize the parameters such as cluster size and time spent by each driver within a cluster that most accurately predicts the risk of infection.

#### 2.4.2. Community risk assessment

**Figure 2:**
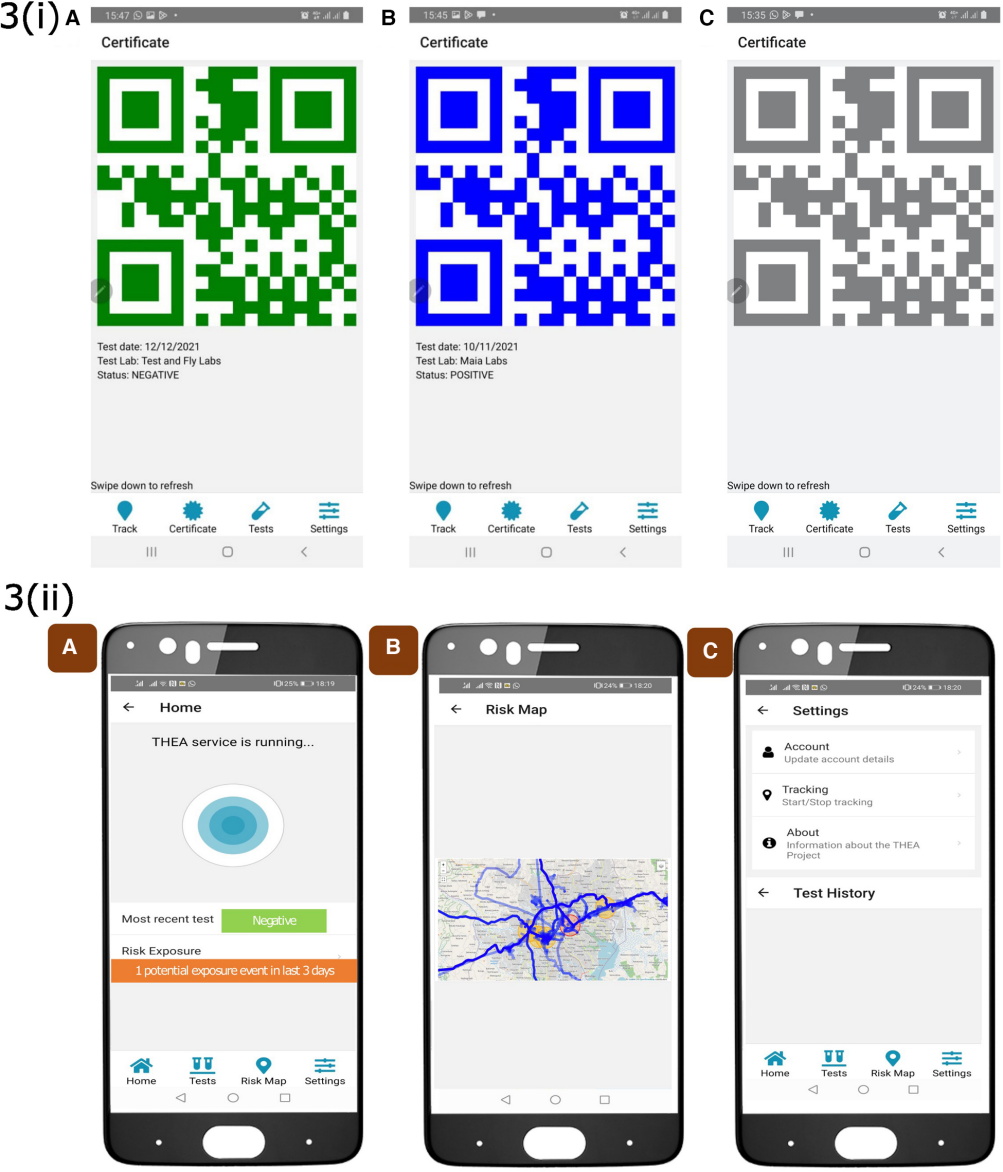
Screen captures of the THEA-mobile phone application. **3(i)** Panel A shows the home page which displays PED data collection, it also shows the most recent result status and the exposure risk. Panel B shows by clicking on the exposure the risk tab, the risk map can be viewed and highlighted in red is the location of the potential exposure event. Panel C is the setting page where the user can manually stop the activity of this application: **3(ii**) Shows the colour-coded QR-codes, A B and C represent negative, positive and invalid test result.

**Figure 3:**
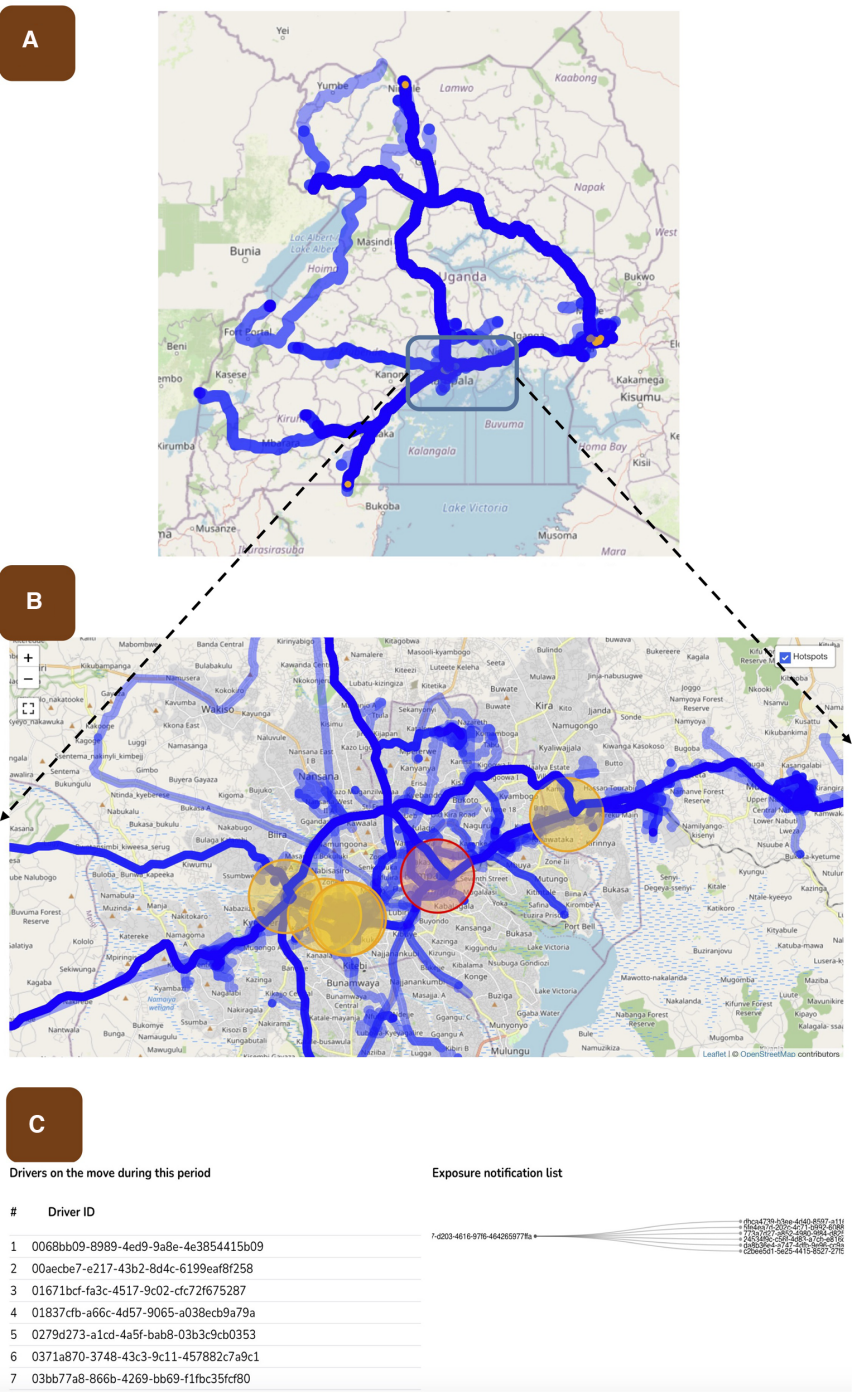
Shows the risk assessment process used to perform contact tracing and associated notifications. (**A**) shows the mapped road network in Uganda using the THEA-GS mobile application and demonstrates the extent of the road network used for testing the tool. The colour intensity of the road network represents the amount of traffic at any given road segment. (**B**) shows the risk analysis map, highlighted in red is the location where a potential transmission event occurred. Orange circles indicate locations where driver congregation for long periods has occurred, but positive cases have not yet been detected. (**C**) shows the logic used to implement the risk assessment. The first column shows all IDs of drivers on the road during a selected period, the second and third column shows the ID of a case and their contacts respectively. It is these contacts that are notified.

The risk assessment engine utilizes the refined proximity estimation data (PED) data from the routing engine and combines it with the diagnostic test information (DTI) using the user’s unique ID. This process follows the methodology described in the proximity estimation section mentioned earlier (11). The risk assessment engine considers the assumption that a potential hotspot will be linked to a community that shares facilities, amenities, or services with truck drivers. The output generated by this component is overlaid onto the road network to create a map highlighting communities at risk. This information is then visualized on the dashboards for public health. An example of the risk assessment conducted in Uganda is illustrated in Figure 2. Additionally, the aggregated GPS data allows for mapping the coverage of the road network, as depicted in Figure 3. This risk mapping process allows public health to evaluate the distribution and impact of risk to the community.

#### 2.4.3. Checking compliance with isolation

Once a driver is notified as positive or potentially exposed, the system initiates a 10-day isolation countdown (which can be adjusted based on local settings). During this period, the unique identifier associated with the driver is not expected to log time-stamped GPS data along the road infrastructure. Compliance with isolation measures is assessed by determining the proportion of notified drivers whose phones did not record any time-stamped GPS data along the road infrastructure within 10 days (approximately 1 and a half weeks) after notification. This metric provides a measure of adherence to the isolation guidelines and helps evaluate the effectiveness of the intervention.

### 2.5. Testing THEA-Gs in Uganda

**Figure 4:**
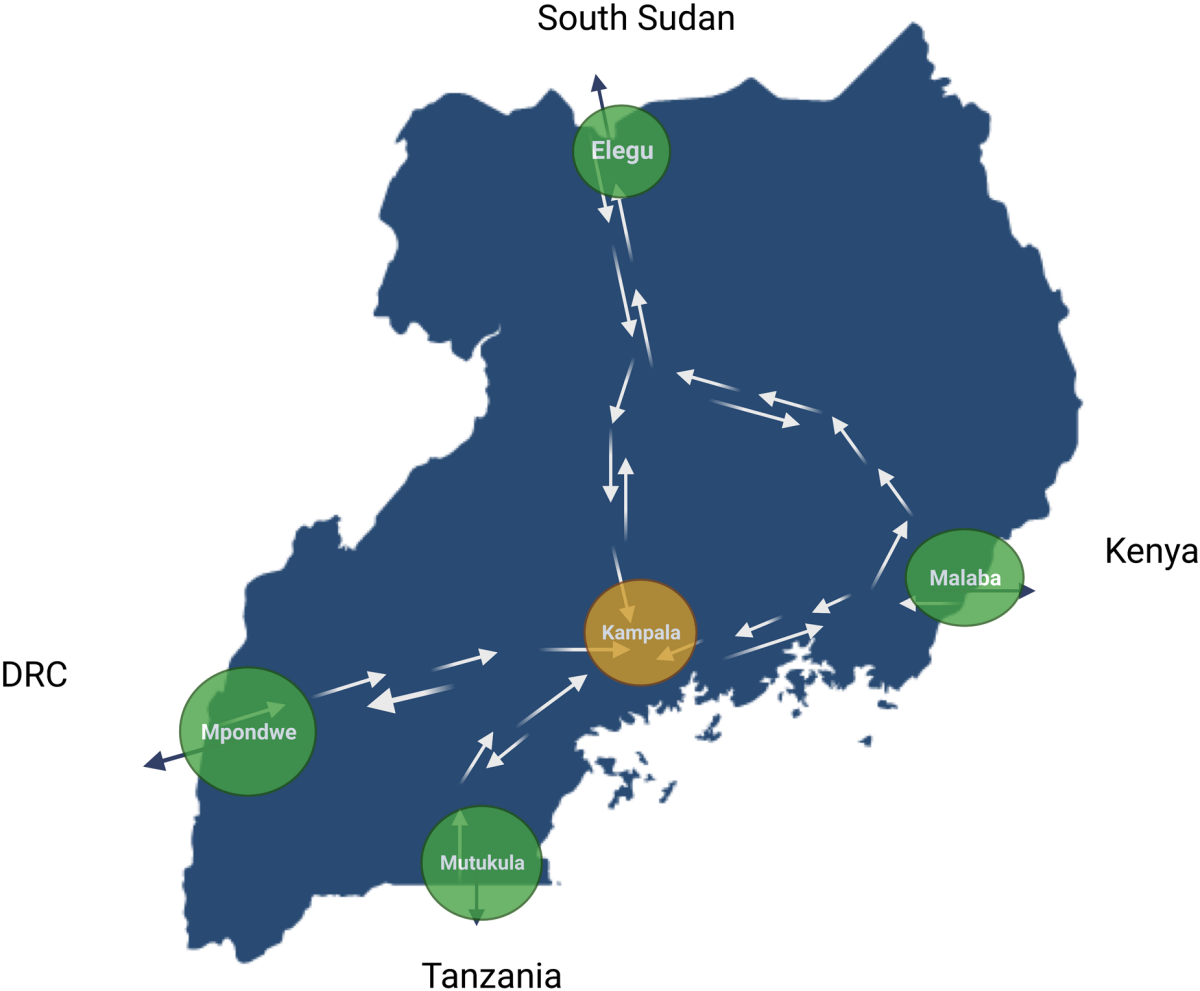
Map showing the location of ports of entry and exit (POEs), including Malaba, Elegu, Mpondwe and Mutukula, at the border with Kenya, South Sudan, Democratic Republic of Congo and Tanzania.

To validate the functionality and effectiveness of THEA-GS, a pilot study was conducted during the Omicron variant wave in Uganda, spanning from October 2021 to October 2022. The pilot study involved several key activities: (a) Integration with Uganda’s result dispatch system (RDS): The tool was integrated with Uganda’s RDS, which is hosted by the Central Public Health Laboratories under the Ministry of Health. This integration facilitated communication and exchange of test results between THEA-GS, the national testing infrastructure and the primary end user. (b) Recruitment of truck drivers at four ports of entry and exit (POEs): Truck drivers at four strategically selected POEs, namely Malaba, Elegu, Mpondwe, and Mutukula, located at the borders with Kenya, South Sudan, Democratic Republic of Congo, and Tanzania, respectively, were enrolled (Figure 4). The recruited drivers downloaded and install the THEA-GS mobile application on their smartphones. Upon downloading the THEA-GS mobile application assigned a unique identifier (UUID) as described in the sections above. By conducting this pilot study, the functionality and efficacy of THEA-GS were assessed in a real-world context, providing valuable insights for further optimization and implementation in public health settings.

To examine the social context of DCT among haulage drivers, eleven focus group discussions (FGDs) were conducted by our social anthropological team. These were done across four ports of entry and two inland haulage stops with significant traffic. These FGDs included truck drivers, union leaders, community members, and local council chairpersons. There were semi-structured with leading questions focusing on attitudes, knowledge and challenges of using DCTs.

### 2.6. Ethical considerations

To test the functionality of this tool, ethical approval was obtained from the School of Public Health Higher Degrees, Research and Ethics Committee at Makerere University (approval number SPH-2021-35) and Uganda National Council for Science and Technology (approval number HS156ES). At the University of Edinburgh, the project host, ethical approval was granted by the Human Ethics and Research Committee at the Easter Bush Campus (HERC_538). Informed consent was obtained for participation in the interviews, focus group discussions, still photography and videography. Data from interviews, questionnaires, GPS, photos, videos and notes were anonymized.

## 3. Results

### 3.1. Testing the functionality of THEA-GS

**Figure 5:**
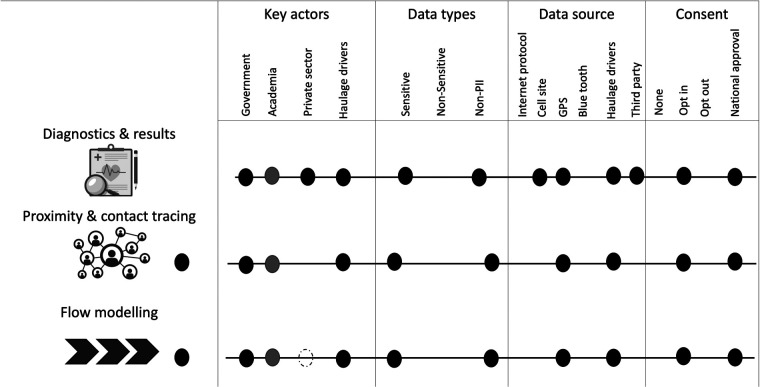
Shows a modified multi-dimensional descriptive topology (16) for THEA-GS. GPS, Global Positioning System; PII, Personally Identifying Information.

THEA-GS is linked via API with the National result dispatch system at the Ministry of Health in Uganda as shown in Figure 1. The results were then sent as a notification to the mobile applications viewed as a colour-coded QR code readable by digital tools at ports of entry and exit (Figures 2, 3). The driver also received an individual risk assessment based on the logic described as described in the methods, and the output is then visualized as a map on the dashboards, this is shown in Figure 3. The collated GPS data also allows us to map the coverage of users on the road network (Figure 4). THEA-GS also uses the same logic to generate a daily risk map of the road network (Figure 4). THEA-GS was developed following the digital framework as shown in Figure 5, and we used the attributes aim to ensure adherence to ethical, social, and legal boundaries for adoption in LMICs (16).

### 3.2. Testing the technical performance of THEA-GS

We tested THEA-GS on ∼105,600 journeys made by 3,270 haulage truck drivers generating 40 million timestamped GPS points along Uganda’s East African road network. Below are some of the technical performances of this tool.

#### 3.2.1. Speed of notification

**Figure 6:**
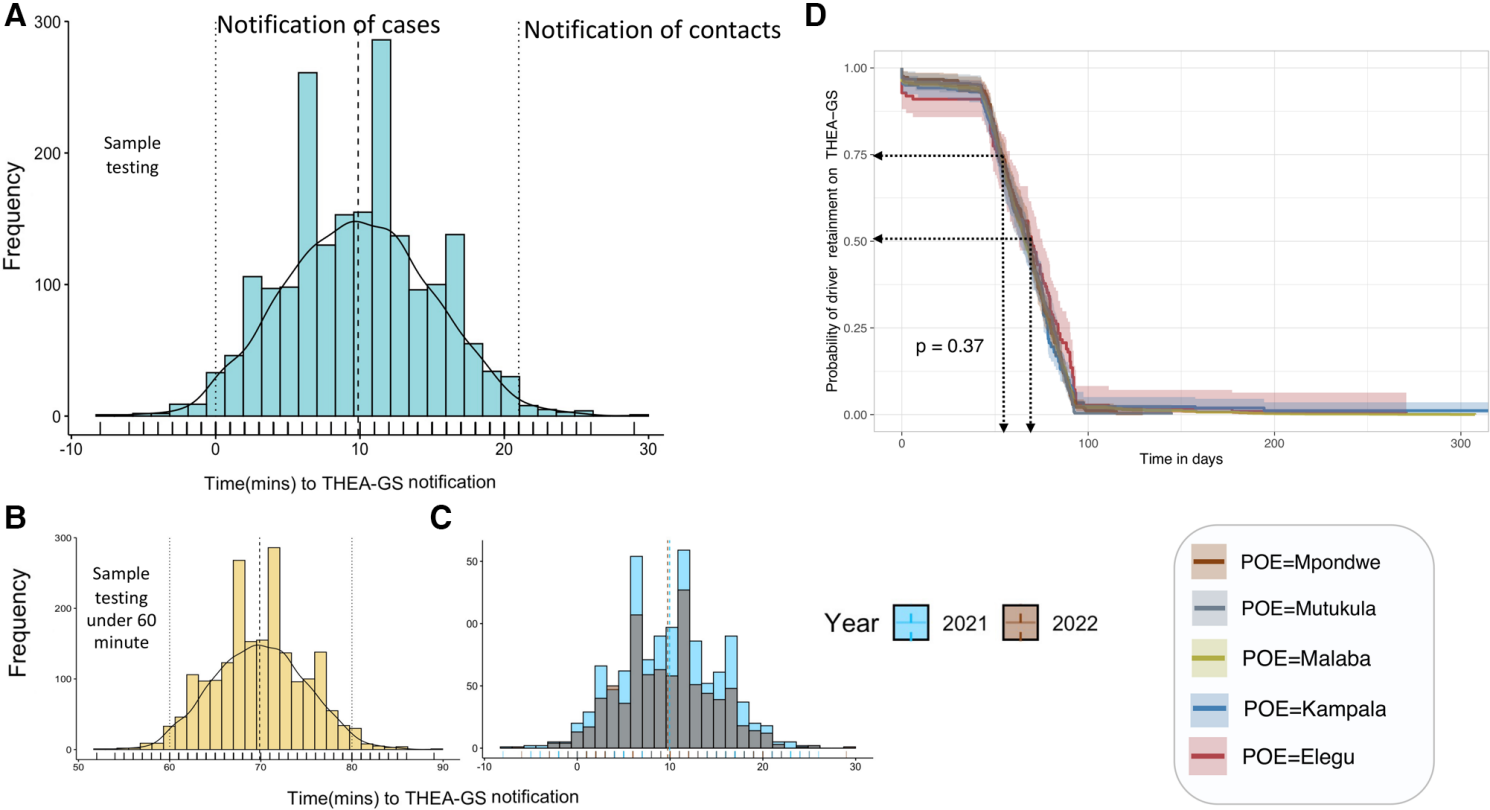
Shows the distribution in the time to notification by the tool (Panel **A**), contrasted by the year (Panel **B**) and the result turn-around (Panel **C**). Panel **D** shows the adherence during the study period.

In Uganda, the THEA-GS server was configured to regularly collect COVID-19 test results from the National Results Dispatch System (RDS) through API integration. The data synchronization occurred every 15 min to ensure the most up-to-date information was available. Considering the time required for laboratory testing of samples, it took an average of 70 min for a case and 80 min for a contact to receive their COVID-19 test results through the THEA-GS smartphone application (Figure 6 and Supplementary Table S2). Therefore on average, it took approximately 10 min (with a range of 1–26 min) to send a COVID-19 test result notification to a case, and 20 min (with a range of 17–40 min) to notify their contacts. Importantly, it is noteworthy that the speed of notification remained consistent over time, as depicted in Figure 6.

#### 3.2.2. Risk assessment

The process of risk assessment and exposure notification within THEA-GS took on average 20 min (with a range of 15–45 min) from the time of case notification. This timeframe encompassed the evaluation of potential risks and the subsequent notification of individuals who may have been exposed to COVID-19. The risk assessment on the mobile phone application was updated once every 24 h, allowing sufficient time for necessary verification by public health authorities (Figures 3, 4). It is important to note that as the tool’s positive predictive value would be expected to improve as the tool is used, the same is true for the time required to post the risk assessment. Furthermore, the hotspots detected by THEA-GS and the infection rate for each day were updated and provided every 24 h (as indicated in Supplementary Table S2).

#### 3.2.3. Compliance and adherence

Out of the 48 cases that were notified, 30 individuals (62.5%) complied with isolation guidelines. This means that 18 individuals (37.5%) did not adhere to isolation measures based on GPS data, which indicated that these drivers continued to travel long distances along the road network within the designated 10-day isolation period. Adherence is defined as the proportion of drivers who remain active on the system throughout the study period, as depicted in Figure 6D. The data shows that there was a high initial adherence rate of 94.5% for the first 45 days (approximately 1 and a half months). However, this rate declined to 50% after 75 days (around 2 and a half months), and this further declines over time. We note that less than 5% of drivers remained on the system beyond 200 days (approximately 6 and a half months). It is worth noting that 5.6% of drivers deleted the application on the same day it was installed.

#### 3.2.4. Challenges with DCT adoption

The focus group discussions (FGDs) conducted during the testing phase of THEA-GS involved 8–12 participants per group, primarily male haulage drivers operating truck routes across Kenya, Uganda, South Sudan, the Democratic Republic of Congo, and Tanzania. While most drivers had some level of awareness about DCT tools, we note that they had a limited understanding of their actual utility and public health benefits. During the FGDs, drivers expressed concerns and apprehension about using THEA-GS, particularly in the first month of testing. The overwhelming concern was the fear of being tracked and the potential of sharing their personal information with the government beyond the project’s duration. In this regard, participants often said “*Your tool may be used by government to spy on us*”. The drivers viewed using a DCT as an additional task with limited immediate benefits, especially considering the unreliable internet connectivity at ports of entry. Additionally, practical challenges such as cracked phone screens, battery issues, incompatible software versions, and a lack of smartphones were highlighted as reasons limiting their participation. In the first week of testing some participants mentioned that “*the tool only works on the latest smartphones*”.

## 4. Discussion

Developing a DCT tool relies on a fine balance between adequate public health benefits, technical functionality and preserving individual privacy. This balance was tested in most countries, especially during the COVID-19 pandemic, where untested ethical frameworks had to be adopted in the name of public health benefit. In reality, without optimizing the balance between ethical, legal and social acceptance, the universal adoption of DCTs for public health will remain challenging. According to the WHO, DCT will play a key role now as countries lift population-wide restrictions amidst persisting incidence of COVID-19 and overstretched public health services (17). The literature suggests that it is still too early to fully assess DCT’s public health impact, and this is not likely to change unless these tools are more widely used (16, 18). To help fill this gap, we have developed a simple, open-source, location-based DCT tool tailored to haulage. We have primarily tested its functionality using 3,270 haulage drivers on the East African road network in Uganda during the Omicron wave of COVID-19. We also report the preliminary performance output and invite other jurisdictions to test its functionality. By doing so we can collectively contribute to the foundation for examining the ethical, legal, and social adoption of such technology for public health in LMICs.

### 4.1. The technical performance of THEA-GS

#### 4.1.1. The speed of contact tracing

The speed and accuracy of DCT is considered crucial parameters for assessing the effectiveness and usefulness of the tool. The key advantage of DCT lies in its ability to leverage mobile phone technologies to significantly enhance the speed and comprehensiveness of contact tracing. Therefore, evaluating these two metrics is essential for justifying the adoption of DCT (19). In Uganda when we compare Manual Contact Tracing (MCT) which took an average of 110 min to DCT. The former included 30 min to obtain a list of contacts via phone call, five minutes for each call made to identified contacts, and an additional five minutes for follow-up with cases and their contacts. These calculations assumed that each case had an average of eight contacts, and the surveillance team worked for twelve hours a day (Supplementary Table S2). During the peak of the COVID-19 pandemic, up to 300 cases were diagnosed daily, requiring 45 person-hours for contact tracing. In contrast, the THEA-GS system notified cases and their contacts within 30 min of posting COVID-19 results in the National Results Dispatch System. This makes DCT approximately 3.6 times faster than MCT per case. Scaling this up to the peak of the pandemic, DCT was approximately 90 times faster than MCT. Although it was technically feasible to update the THEA-GS system with positive test results as soon as they were recorded, we opted to access the Result Dispatch System (RDS) at specified time intervals (every 15 min) to avoid unnecessary strain on the testing database infrastructure.

#### 4.1.2. Effectiveness and benefit to public health

The jury is still out on the effectiveness and the public health benefit (16, 18). This is because, some studies have shown a high DCT efficacy of 80% with up to 11% discovery of close contacts that would not have otherwise been identified using MCT (20). On the other hand, others have shown that DCT did not offer sufficient public health benefit to populations during the pandemic (21). Therefore, more studies are needed to estimate both the effectiveness and public health benefit to cement the role of these tools in the epidemiological toolkit for controlling infectious disease outbreaks. Our study does not offer an immediate solution to this challenge but provides an open-source tool for countries to test during disease outbreaks where haulage is a risk group. The challenge seems to be the low uptake, which means the minimum population threshold to assess public health utility is rarely met (21–23). In Uganda, only ∼4% (*n* = 3,270) of the total haulage driver population was enrolled, which precludes our ability to evaluate the efficiency and public health utility.

Parameters such as the positive predictive value (PPV), which is the proportion of notified contacts that eventually test positive within a specific timeframe, could have been used to evaluate the accuracy of THEA-GS. However, the limited sample size of the pilot study precludes our ability to robustly calculate this value. While the availability of tests targeted at haulage drivers was sufficient, we faced challenges persuading the drivers to adopt and utilize the tool, due to the time it took to establish trust and engagement. By the time trust was established, the peak of the Omicron phase had passed and the numbers of positive cases had dropped below the levels required for evaluation of the PPV. It is also important to note that the optimal utility of Digital Contact Tracing (DCT) is achieved at the start of an outbreak when community transmission is low and only a few transmission chains exist. Despite the relatively low number of cases after the Omicron wave, the level of community transmission was still high enough that it was more likely for notified contacts to be infected in the community outside the predicted hotspots by unrelated transmission chains. We expect that further uptake of this tool in more ideal settings will be important in the evaluation of its accuracy and effectiveness.

#### 4.1.3. Transparency

As developers of DCT tools we bear the responsibility of ensuring the safety and reliability of the tools. In reality, safety, be that perceived or real, influences the trust of the target population and government institutions (2, 21). Here, we have taken steps to foster trust by promoting transparency, i.e., we have adopted an open-source approach for our tool and the supporting code. By making the code publicly accessible, we encourage public scrutiny and enable a broader user base to examine and contribute to the technology. This commitment to openness allows for continuous development, improvement, and protection of the rights of end users.

THEA-GS is a place-based DCT tool using timestamped GPS data instead of Bluetooth Low Energy (BLE) to avoid any reliance on proprietary protocols such as Google and Apple’s Exposure Notification (GAEN) system widely used by other DCTs (24). This also avoids the shortcomings of Bluetooth technology where the signal strength and obstacles between these devices can affect the accuracy of proximity detection. Here we refine the utility of GPS by referencing ground truth information (GTI), also referred to as points of interest (POI) tracking. This allows haulage-relevant features such as traffic alerts to be incorporated into the app to not only enhance functionality but contribute to safety on the road network. To the best of our knowledge, this is the first open-source DCT tool tailored to haulage. Examples of digital tools that have been used with haulage are ArriveCAN and RECDTS (Regional Electronic Cargo and Driver Tracking System), used in Canada and East Africa, respectively. However, both are closed-source tools (25, 26). Importantly, neither ArriveCAN nor RECDTS are DCT tools as they only notify the drivers of a positive test but not their potential contacts (Table 2). Most of the other open-source DCT tools used for the general population currently under active development include Coronamelder (Netherlands), Corona-Warn-App (Germany), Immuni App (Italy), NHS COVID-19 App (UK) and the TousAntiCovid (France). All these tools use the BLE to determine proximity-based tracing. Beyond targeted DCT tools, most population-wide tools used in Europe and Asia, are not actively being developed as open-source.

**Table 2:** List of applications and tools that have been applied to the haulage sector.

^1^Name	Features	Proximity tracking approach	Link
ArriveCAN/CovidAlert		Google/Apple Exposure Notification (GAEN)	https://www.canada.ca/en/border-services-agency/services/arrivecan.html
RECDTS	Test result notification		https://innov.afro.who.int/emerging-technological-innovations/regional-electronic-cargo-and-driver-tracking-system-recdts-2758
NHS COVID-19 UK		GAEN	https://github.com/nihp-public/covid19-app-system-public
Quarano			https://github.com/quarano/quarano-application

^1^ It is worth noting that none of these tools are DCTs specifically tailored to haulage truck drivers.

DCTs, digital contact tracing tools

### 4.2. Efforts to safeguard individual privacy and universal access

#### 4.2.1. Privacy and security

Unlike Bluetooth methods, GPS-based methods carry the risk of potentially revealing detailed individual movements (16, 27). To mitigate this risk, we have implemented measures to pseudo-anonymize the data, ensuring that personal identifying information is not used. Furthermore, any identifiable information that is collected, such as mobile phone numbers, undergoes encryption using the Advanced Encryption Standard (AES) with 128 bits. Access to this information is strictly limited to approved users, and all access attempts are logged for transparency and security purposes. It is important to note that THEA-GS is designed to track users only when they are located on designated road infrastructure. This deliberate limitation ensures that the tracking is focused solely on the occupational activities of the drivers and does not extend to their private activities. To uphold individual autonomy, primary end-users have the freedom to opt out of any of the processes at any time. Detailed instructions for opting out are provided in the accompanying online documentation, enabling users to make informed decisions and have control over their participation in the system (https://project-thea.github.io/thea-gs/).

#### 4.2.2. Digital inclusion and equity

While internet access is widespread in most developed countries, this is not the case in many countries, which can limit the usefulness of most DCT tools. However, THEA-GS is specifically designed to address this limitation by collecting timestamped GPS data even when the user’s phone is offline and by improving the accuracy of these data points using a routing model on the THEA-GS server. Furthermore, our application is designed to be compatible with popular versions of the Android smartphone operating system, including version 5 and later. This covers over 80% of Android users in Africa (Mobile Operating System Market Share Africa, n.d.). In contrast, many DCT tools developed in Europe and Asia are only compatible with Android OS versions 10 or higher, which would exclude a significant portion of Android users in Africa (28). We have also developed and submitted the iOS version of the application pending approval by the Apple App Store. Additionally, our tool is adaptable to different computing infrastructures, allowing countries with limited computing capacity to adjust the system’s parameters. This flexibility enables them to reduce the computational load by adjusting the frequency of notifications or the extent of the road network analysis. In Uganda, we developed THEA-GS in collaboration with the Ministry of Health of Uganda using a centralized data model for storage and processing to ensure good data governance.

### 4.3. Challenges with uptake during testing of THEA-GS in Uganda

In Uganda, the main implementation challenges were internet connectivity, battery life and privacy concerns. Previous eHealth studies have shown that incentives such as subsidized airtime are effective in increasing the rate of participation (29). Although most ports of entry and exit (POE) had free internet provided by the government via the National Information Technology Authority (NITA), it wasn’t always available. The POE are a critical point for the THEA-GS because this is where the testing and onboarding were done. Therefore, sustainable deployment of this tool may require incentives that promote uptake, such as the Internet.

In Uganda, the uptake of THEA-GS during the testing phase faced several challenges, including issues related to internet connectivity, battery life, and privacy concerns. Previous eHealth studies have used incentives, such as subsidized airtime to promote participation rates (29). However, the sustainability of such a model beyond the project remains questionable. The government of Uganda has had initiatives to provide free internet access through the National Information Technology Authority (NITA) at the ports of entry and exit, but this service was not always available.

Data security, privacy, and confidentiality protection were important concerns raised by stakeholders in previous studies conducted in Uganda (30). Here we encountered resistance from stakeholders regarding data protection, particularly in terms of guaranteeing anonymity and addressing concerns about supervisors or managers having access to their daily movements. We noted that over time our awareness campaigns through stakeholder engagement meetings increased their engagement. However as shown by our adherence statistics, keeping drivers engaged and using the DCT likely requires much more than just awareness.

Although challenging translating material into local languages such as Swahili improved engagement with the tool and is one of the aspects the team will pursue further. Indeed this has been reported by other studies examining the willingness to use COVID-19 tracing tools where an effective communication strategy is used to disseminate knowledge, promote understanding, and prevent misinformation that could adversely affect uptake (31). Beyond misconceptions, the adherence result in Figure 6D shows an inevitable attrition if the tool’s functionality is restricted to public health use. Therefore, it is worthwhile exploring other sector-specific functionality such as road safety, journey planning and security alert functionalities to keep the primary stakeholders engaged.

### 4.4. The potential utility of THEA-GS beyond public health

COVID-19 is not the first and certainly will not be the last infectious disease to threaten the haulage sector in the global south. Therefore, THEA-GS can also be implemented during outbreaks of other diseases such as Ebola, Marburg, and Congo Crimean Fever. With minor modifications, the tool can also be used for tracking the movement of animals transported by haulage, to provide vital contact structure information that can be used to control economically important livestock infectious diseases such as Foot and Mouth disease and African Swine fever whose long-distance transmission is attributable to haulage transportation (32, 33). By including other safety features, such as road hazard alerts, and convenience features, such as real-time traffic congestion, the tool can also be used to improve road safety and provide additional value for users in the haulage sector.

### 4.5. Limitations of study

It is important that DCTs are integrated into a comprehensive network of mass testing and ensure high levels of participation and compliance with self-isolation measures among the target population. These elements are essential for accurately assessing the utility and impact of such tools. During our testing phase in Uganda, we encountered logistical challenges that limited the reach of our testing efforts, resulting in only approximately 4% of the target population being involved. As a result, we acknowledge that we could not reliably evaluate the overall utility and impact of the tool within the population. However, we have provided a comprehensive report on the functionality of the tool. This includes detailed information on its features, capabilities, and technical performance, offering valuable insights into its potential effectiveness and use in a broader context.

## 5. Conclusions

In conclusion, we show that consultative implementation of a DCT tool with haulage drivers as primary stakeholders is important as part of the trust-building activities. The discussion with drivers highlighted apprehensions about the use of digital tools which should be addressed. The tool we have developed is designed for use by public health institutions with the mandate for contact tracing and requires minimum infrastructure requirements.

We also show the need to document ethical, legal, and social constraints of using DCT tool as it will help generate evidence to guide governance and oversight. When we conducted this testing in Uganda, we experienced a vacuum in guidelines around the use of digital contact tracing especially for the haulage sector. Attrition from such systems is inevitable without incentives to keep primary stakeholders engaged with the tool; we highly recommend incorporating incentives such as traffic reports, hands-free peer-to-peer communication, security alerts and other products for sustainable adherence.

THEA-GS to date is the only open-source digital contact tracing tool tailored to the haulage sector in Africa. While we have successfully tested its functionality in Uganda, we acknowledge that a comprehensive evaluation of its effectiveness and public health benefits was not fully realized due to challenges related to adoption and participation. Therefore, by making it open source, we invite other countries to test and implement it in their relevant contexts to allow for a collective of evaluating its utility on a broader scale.

## Data Availability

The datasets presented in this article are not readily available due to local legal, ethical and privacy restrictions. Requests to access the datasets be directed to adrian.muwonge@roslin.ed.ac.uk.

## References

[1] WHO. Global strategy on digital health 2020–2025. Geneva: World Health Organization. Available at: https://apps.who.int/iris/handle/10665/344249 (Accessed October 12, 2022). (2021).

[2] Blasimme A Ferretti A Vayena E. Digital contact tracing against COVID-19 in Europe: current features and ongoing developments. Front Digit Health. (2021) 3:2–4. 10.3389/fdgth.2021.660823 PMC8521942 34713135

[3] Niakan Kalhori SR Bahaadinbeigy K Deldar K Gholamzadeh M Hajesmaeel-Gohari S Ayyoubzadeh SM. Digital health solutions to control the COVID-19 pandemic in countries with high disease prevalence: literature review. J Med Internet Res. (2021) 23:2–7. 10.2196/19473 PMC7951053 33600344

[4] WHO. Digital tools for COVID-19 contact tracing: Annex: contact tracing in the context of COVID-19, 2 June 2020. Geneva, Switzerland: World Health Organization (2020).

[5] Lewis D. Why many countries failed at COVID contact-tracing — but some got it right. Nature. (2020) 588:384–7. 10.1038/d41586-020-03518-4 33318682

[6] Al Musleh AA Asim M Abdurahiman S El-Menyar A Khan NA Al-Thani H. Bio-secure bubble during the COVID-19 pandemic to host the Asian football confederation (AFC) champions league: a retrospective observational study. Health Sci Rep. (2023) 6:e985. 10.1002/hsr2.985 36514329 PMC9732739

[7] Bajunirwe F Izudi J Asiimwe S. Long-distance truck drivers and the increasing risk of COVID-19 spread in Uganda. Int J Infect Dis. (2020) 98:1–4. 10.1016/j.ijid.2020.06.085 32615323 PMC7323644

[8] Kagucia EW Gitonga JN Kalu C Ochomo E Ochieng B Kuya N Anti-severe acute respiratory syndrome coronavirus 2 immunoglobulin G antibody seroprevalence among truck drivers and assistants in Kenya. Open Forum Infect Dis. (2021) 8:ofab314. 10.1093/ofid/ofab314 34660838 PMC8519263

[9] Muwonge A Mpyangu CM Nsangi A Mugerwa I de Bronsvoort BMC Porphyre T Developing digital contact tracing tailored to haulage in East Africa to support COVID-19 surveillance: a protocol. BMJ Open. (2022) 12:e058457. 10.1136/bmjopen-2021-058457 36691163 PMC9441735

[10] Valhalla/valhalla. (2023). Available at: https://github.com/valhalla/valhalla (Accessed March 9, 2023).

[11] Yuan J Zheng Y Zhang C Xie XX Sun GZ Du M Map-matching for low-sampling-rate GPS trajectories. GIS Proc ACM Int Symp Adv Geogr Inf Syst. (2020) 433–434:1–3. 10.1016/j.ins.2017.12.031

[12] Laravel/jetstream. (2023). Available at: https://github.com/laravel/jetstream (Accessed March 9, 2023).

[13] Curry DM. An algorithm for clustering animals by species based upon daily movement. Procedia Comput Sci. (2014) 36. 10.1016/j.procs.2014.09.066

[14] Abdullah D Susilo S Ahmar AS Rusli R Hidayat R. The application of K-means clustering for province clustering in Indonesia of the risk of the COVID-19 pandemic based on COVID-19 data. Qual Quant. (2022) 56:1283–91. 10.1007/s11135-021-01176-w 34103768 PMC8173859

[15] Wang H Ou J Yuan Y. Strategy of data processing for GPS rover and reference receivers using different sampling rates. IEEE Trans Geosci Remote Sens. (2011) 49:1144–49. 10.1109/TGRS.2010.2070509

[16] Gasser U Ienca M Scheibner J Sleigh J Vayena E. Digital tools against COVID-19: taxonomy, ethical challenges, and navigation aid. Lancet Digit Health. (2020) 2:e425–9. 10.1016/S2589-7500(20)30137-0 32835200 PMC7324107

[17] World Health Organization Regional Office for the Western Pacific. Selecting digital contact tracing and quarantine tools for COVID-19 : Guiding principles and considerations for a stepwise approach. Manila, Phillipines: WHO Regional Office for the Western Pacific (2020).

[18] Hossain AD Jarolimova J Elnaiem A Huang CX Richterman A Ivers LC. Effectiveness of contact tracing in the control of infectious diseases: a systematic review. Lancet Public Health. (2022) 7:e259–71. 10.1016/S2468-2667(22)00001-9 35180434 PMC8847088

[19] Chen AT-Y Thio KW. Exploring the drivers and barriers to uptake for digital contact tracing. Soc Sci Humanit Open. (2021) 4:1–10. 10.1016/j.ssaho.2021.100212 PMC8494623 34642660

[20] Elmokashfi A Sundnes J Kvalbein A Naumova V Reinemo S-A Florvaag PM Nationwide rollout reveals efficacy of epidemic control through digital contact tracing. Nat Commun. (2021) 12:5918. 10.1038/s41467-021-26144-8 34635661 PMC8505561

[21] Vogt F Haire B Selvey L Katelaris AL Kaldor J. Effectiveness evaluation of digital contact tracing for COVID-19 in New South Wales, Australia. Lancet Public Health. (2022) 7:e250–6. 10.1016/S2468-2667(22)00010-X 35131045 PMC8816387

[22] Braithwaite I Callender T Bullock M Aldridge RW. Automated and partly automated contact tracing: a systematic review to inform the control of COVID-19. Lancet Digit Health. (2020) 2:e607–e621. 10.1016/S2589-7500(20)30184-9 32839755 PMC7438082

[23] Rowe F. Contact tracing apps and values dilemmas: a privacy paradox in a neo-liberal world. Int J Inf Manag. (2020) 55:102178. 10.1016/j.ijinfomgt.2020.102178 PMC7324927 32836636

[24] Exposure Notifications: Helping fight COVID-19—Google. Expo. Notif. Help. Fight COVID-19—Google. (n.d.). Available at: https://www.google.com/intl/en_us/covid19/exposurenotifications/ (Accessed April 3, 2023).

[25] Agency CBS. Use ArriveCAN for a faster border experience. (2022). Available at: https://www.canada.ca/en/border-services-agency/services/arrivecan.html (Accessed April 3, 2023).

[26] RECDTS Driver App—Apps on Google Play. (n.d.). Available at: https://play.google.com/store/apps/details?id=bsmart.technology.recdts.da&hl=en (Accessed April 3, 2023).

[27] Bengio Y Janda R Yu YW Ippolito D Jarvie M Pilat D The need for privacy with public digital contact tracing during the COVID-19 pandemic. Lancet Digit Health. (2020) 2:e342–4. 10.1016/S2589-7500(20)30133-3 32835192 PMC7266569

[28] Dowthwaite L Fischer J Vallejos EP Portillo V Nichele E Goulden M Public adoption of and trust in the nhs COVID-19 contact tracing app in the United Kingdom: quantitative online survey study. J Med Internet Res. (2021) 23:2–15. 10.2196/29085 PMC8451731 34406960

[29] Gibson DG Wosu AC Pariyo GW Ahmed S Ali J Labrique AB Effect of airtime incentives on response and cooperation rates in non-communicable disease interactive voice response surveys: randomised controlled trials in Bangladesh and Uganda. BMJ Glob Health. (2019) 4:e001604. 10.1136/bmjgh-2019-001604 31565406 PMC6747927

[30] Mwaka E Nakigudde J Ali J Ochieng J Hallez K Tweheyo R Consent for mobile phone surveys of non-communicable disease risk factors in low-resource settings: an exploratory qualitative study in Uganda. mHealth. (2019) 5:26. 10.21037/mhealth.2019.07.05 31559271 PMC6737387

[31] Kasozi KI MacLeod E Ssempijja F Mahero MW Matama K Musoke GH Misconceptions on COVID-19 risk among Ugandan men: results from a rapid exploratory survey, April 2020. Front Public Health. (2020) 8:416. 10.3389/fpubh.2020.00416 32850606 PMC7405654

[32] Walz E Middleton J Sampedro F VanderWaal K Malladi S Goldsmith T. Modeling the transmission of foot and mouth disease to inform transportation of infected carcasses to a disposal site during an outbreak event. Front Vet Sci. (2020) 6:1–6. 10.3389/fvets.2019.00501 PMC6971117 31993448

[33] Neumann E Hall W Dahl J Hamilton D Kurian A. Is transportation a risk factor for African swine fever transmission in Australia: a review. Aust Vet J. (2021) 99:459–68. 10.1111/avj.13106 34235721

