# Supplementary material for: An open-source digital contact tracing system tailored to haulage
Source: Front Digit Health. 2023 Jul 19;5:1199635. doi: 10.3389/fdgth.2023.1199635 (PMC10394895; doi:10.3389/fdgth.2023.1199635)
Supplement: Supplementary file 1 [file Table1.docx]

Supplementary Material

**Table S1**: This is a list of common DST features and variables collected, we highlight the featured that have been included in THEA-GS for the digital contact tracing utility. We have also proposed features that can be included to improved adherence

| **Features** | **Included** | **Comments** |
| --- | --- | --- |
| Personal data |  | No personal data is collected, other than the phone number |
| Test results | √ | Test result notification are sent as shown in **Figure 3A** |
| Test history | √ | Test history collated by app as shown in **Figure 3C** |
| Identification |  | Not collected |
| Demographics |  | Not collected |
| Phone number | √ | Collected but encrypted at capture |
| Symptoms |  | Not collected |
| Travel history | √ | Tracking data is restricted to the Road infrastructure |
| Vaccination history |  | Not yet included but should be included for adherence |
| Risk profile (Individual) | √ | Computed and accessible on the app **Figure 3B** |
| Bluetooth access |  | Not applicable |
| Digital consent | √ | Mandatory before installation of the application |
| **Functionality** |  |  |
| GPS-Proximity | √ | Timestamped GPS used |
| Order test |  | Not included |
| Count down quarantine time | √ | Part of the Geofencing module but not active |
| QR-code scan | √ | Included for recruitment at POEs |
| ID scan |  | Can be included in the future |
| Risk profile (road network) | √ | Computed and accessible via the mobile app (**Figure 3B**) |
| **Governance** |  |  |
| Web-portal strictly for public health | √ | The use of this tool is limited to National public health institutions |

**Table S2**: The parameters generated by THEA-GS system are used to evaluate the speed of digital contact tracing. These are contrasted against the time it takes to implement the same step in the manual contact tracing. It is noteworthy that the frequency recording these parameters are adjustable to fit the local context

| **Parameter** | **Speed** | **Comments** |
| --- | --- | --- |
| *Results notification* | THEA-GS: On average 10 minutes after result posting in RDS  MCT: ~ **30** minutes | The driver receives a notification 70 minutes from sample collection. MCT contacts was varied, by phone call or SMS |
| *Risk Assessment* & contact list generation | THEA-GS - Instant  MCT: on average **30** minutes | The risk assessment is computed instantly by THEA-GS but the hotspot map posted every 24 hours. |
| *Notifying contacts* | THEA-GS: 20 minutes after the results of a case posted in RDS.  MCT: ~**90**-180 minutes after the results of a case posted in RDS | Whenever a positive test was detected, an exposure notification list is generated instantly for THEA-GS, then a notification sent to contacts |
| *Follow-up* | THEA-GS: Notification every 36 hours to report changes in signs  MCT: Follow up call supposed to be made 72-96 hour | MCT- this was not always done |
| *Compliance* | THEA-GS: geofencing activates after first result and contact notification  MCT: difficult to evaluate adherence | THEA-GS: If a notified driver was on the road within 14 days of notification, they were considered noncompliant |

**Table S3**: Shows preliminary THEA-GS performance summary from data collected from truck driver data during the test period of the tool in Uganda. Some of the data on our tool is contrasted against the RDS to assess the level of coverage and concordance

| **Performance variable** | **Statistics** | **Comments** |
| --- | --- | --- |
| Total drivers | 3270 | This represents ~28% of driver data* in the RDS between December 2021 and September 2022 |
| Total tests | 3800 | This represents 16% of tests for all truck drivers in RDS between December 2021 and September 2022  THEA-GS mean test rate = 11.4/day  RDS mean test rate = 68/day |
| Tested Positives | 48 | THEA-GS positivity rate = 1.3%  RDS* positivity rate= 3.5%  (1330/38126) |
| Positives with tracking data | 20 | Note that the system collects historical test results, so it is possible that a person tested positive prior to joining the system. |
| Positives with GPS data | 28 | This the number of positive cases used to test the risk assessment functionality |
| Total contact notification list size | 125 | This translates to 1:4 case to a suspected contact ratio |
| Noncompliance with notification | 16% | 20/125 did not isolate when they received a notification |
| Compliance with notification | 84% | 105/125 isolated when they received a notification |
| Request per second | 1000/s |  |
| Executed tasks per hour | 420 | On average 360 requests processing jobs and 60 routing engine jobs in an hour |
| Data points collected | 40 million | On average 3.45 million timestamped GPS were collected per month |
| Adherence | 94.5% for 45 days, 50% for 75 days | The proportion of the drivers retained on the system after a specific period. Interestingly on 5.6% delete the application on the day of installation |

* There are a total of 38,126 COVID-19 tests results in the National Results Dispatch System (RDS) recorded under truck drivers during this period, which means our study represents ~ 10% of test results from this group.
